# Long Noncoding RNA Can Be a Probable Mechanism and a Novel Target for Diagnosis and Therapy in Fragile X Syndrome

**DOI:** 10.3389/fgene.2019.00446

**Published:** 2019-05-22

**Authors:** Ge Huang, He Zhu, Shuying Wu, Manhua Cui, Tianmin Xu

**Affiliations:** The Second Hospital of Jilin University, Changchun, China

**Keywords:** long noncoding RNA, fragile X syndrome, fragile X-related primary ovarian insufficiency, fragile X-associated tremor/ataxia syndrome, FMR4, FMR6, BC1, TUG1

## Abstract

Fragile X syndrome (FXS) is the most common congenital hereditary disease of low intelligence after Down syndrome. Its main pathogenic gene is fragile X mental retardation 1 (FMR1) gene associated with intellectual disability, autism, and fragile X-related primary ovarian insufficiency (FXPOI) and fragile X-associated tremor/ataxia syndrome (FXTAS). FMR1 gene transcription leads to the absence of fragile X mental retardation protein (FMRP). How to relieve or cure disorders associated with FXS has also become a clinically disturbing problem. Previous studies have recently shown that long noncoding RNAs (lncRNAs) contribute to the pathogenesis. And it has been identified that several lncRNAs including FMR4, FMR5, and FMR6 contribute to developing FXPOI/FXTAS, originating from the FMR1 gene locus. FMR4 is a product of RNA polymerase II and can regulate the expression of relevant genes during differentiation of human neural precursor cells. FMR5 is a sense-oriented transcript while FMR6 is an antisense lncRNA produced by the 3′ UTR of FMR1. FMR6 is likely to contribute to developing FXPOI, and it overlaps exons 15–17 of FMR1 as well as two microRNA binding sites. Additionally, BC1 can bind FMRP to form an inhibitory complex and lncRNA TUG1 also can control axonal development by directly interacting with FMRP through modulating SnoN–Ccd1 pathway. Therefore, these lncRNAs provide pharmaceutical targets and novel biomarkers. This review will: (1) describe the clinical manifestations and traditional pathogenesis of FXS and FXTAS/FXPOI; (2) summarize what is known about the role of lncRNAs in the pathogenesis of FXS and FXTAS/FXPOI; and (3) provide an outlook of potential effects and future directions of lncRNAs in FXS and FXTAS/FXPOI researches.

## Introduction

Fragile X syndrome (FXS) is a congenital hereditary disease associated with low intelligence, and is second common only to Down syndrome. The main pathogenic gene of the fragile X syndrome (FXS) is the fragile X mental retardation 1 (FMR1) gene located at Xq27.3, and it was first cloned in 1991 by Verkerk et al. (1991). Between Xq27 and Xq28, the chromosomes are abnormally concentrated during meiosis, forming an extremely fragile filamentous site. Therefore, this syndrome is regarded as a fragile X chromosome syndrome. As well, the 5′ end untranslated region (UTR) of the gene has a highly conserved CpG island with a length of 250 bp, which includes a trinucleotide repeat expansion (CGG)n whose sequence is abnormally amplified and methylated in individuals with fragile X chromosome syndrome (Bardoni and Mandel, 2002). Based on the CGG trinucleotide repeat numbers, the sequence is divided into full mutation, permutation, gray zone, and normality (Allingham-Hawkins et al., 1999; Bardoni and Mandel, 2002). In addition, full mutation, an expansion beyond 200 repeats, is associated with typical clinical symptoms of FXS which is responsible for intellectual disability, autism, and so on. Besides, permutation which corresponds to 55–200 repeats may contribute to development of fragile X-related primary ovarian insufficiency (FXPOI) and the fragile X-associated tremor/ataxia syndrome (FXTAS). A size of 45–54 is known as a gray zone, while normality corresponds to 5–44 repeats (Wittenberger et al., 2007; Ciaccio et al., 2017).

There is a lot of evidence that all proteins originate from only 1.2% of the genome, although 40% or higher of the human genome is transcribed into RNA (Carninci et al., 2005; Cheng et al., 2005; Consortium et al., 2007), known as non-protein-coding RNA (ncRNA) and enriched in the brain (Djebali et al., 2012). As well, ncRNAs are classified into different categories based on their functions and sizes. For instance, ncRNAs are differentiated into short and long noncoding RNAs (lncRNAs). Also, we introduced the long noncoding RNAs (lncRNAs) in this article, having a wide range of functions. They are also a kind of ncRNA with a length of more than 200 nucleotides while short noncoding RNAs certainly have less than 200 nucleotides. Short noncoding RNAs include microRNAs (miRNAs), small-interfering RNA (siRNA), and piwi-interacting RNA (piwi-RNA) and small nuclear RNAs (snRNA) (Zhou et al., 2019). For example, miRNAs are noncoding single-stranded RNAs consisting of 18–25 nucleotides in length (Akbari Kordkheyli et al., 2019). Previous studies have recently indicated that lncRNAs contribute to the pathogenesis of both the full mutation and premutation carriers, especially the nervous disorders. Still, there is no summary of function and mechanism of lncRNAs in FXS patients and premutation carriers, while the article sums up and explains the link between FXS and lncRNAs in detail.

## Functions and Mechanisms of Long Noncoding RNAs

NcRNAs play a significant role in human diseases. It is also demonstrable that ncRNAs have multiple functions. For example, pi-RNAs can repress translation (Fire et al., 1998; Carmell et al., 2007; Houwing et al., 2007), miRNAs can inhibit translation, while siRNAs can lead to silencing of a wide range of genetic targets and degrade mRNA (Fire et al., 1998; Dana et al., 2017). And a lot of evidence implicates that numerous protein-coding genes such as FMR1 have antisense transcripts. In some instances, antisense transcription manipulation may result in sense transcription inhibition or make sense transcription more stable. These phenomena are known as discordant regulation and concordant regulation, respectively (Katayama et al., 2005). However, it is still not certain whether the exact regulation mechanisms of sense transcriptions are regulated by antisense partners, which may be various and complicated; thus, there is a need for more and further studies. Previous researches have revealed that the expression pattern of ncRNAs has changed in many patients suffering from human disorders, such as cardiovascular disease, neuronal dysfunction, and cancer (Haemmig and Feinberg, 2017; Nicolas, 2017; Cai et al., 2019; Shao et al., 2019; Zhang et al., 2019). They all indicate that ncRNAs can be functionally related to human diseases. Therefore, ncRNAs are probably taken for potential drug targets (Khalil et al., 2008; Nicolas, 2017). Short noncoding RNAs have been extensively explored and reviewed that they tend to affect the gene expression through the interference with translation or posttranscriptional mechanisms (Rother and Meister, 2011; Ojha et al., 2019). However, we only learned in recent years that lncRNAs can contribute to regulating cellular functions and/or physiology as regulatory factors. As the function of the lncRNAs gradually surfaces, lncRNAs have attracted more and more attention as potential biomarkers and/or drug targets (Kung et al., 2013; Tang et al., 2014; Zhu et al., 2018; Zou et al., 2018).

In particular, lncRNAs can originate from both sense and antisense chains of genes that can encode proteins. As well, they are likely to be a transcript of promoter, intron, and 3′ end region. More specifically, lncRNAs are grouped into five categories according to the nearest protein-coding genes, which are sense, antisense, bidirectional, intronic, and intergenic lncRNAs. Besides, protein-coding genes are defined as the sense DNA. Transcriptions of sense lncRNAs incline to the occurrence of the sense DNA strand, which is different in the antisense lncRNAs. However, they all overlap one exon or more. Bidirectional lncRNAs are transcribed from the promoter in two directions, and their length is usually several hundred base pairs (bps) (Sarfi et al., 2019). Intronic lncRNAs do not overlap any exons and are the transcripts that originate from introns in any directions. Meanwhile, intergenic lncRNAs are stand-alone, meaning that they can exist in the sequence space without contain protein-coding genes, also known as large intergenic (or intervening) ncRNAs (Kung et al., 2013; Kumar and Goyal, 2017). At the same time, lncRNAs can interact with DNA, RNA, and protein, and other biological macromolecules and perform many biological functions such as regulation of the activity of transcription and the epigenetic landscape of their original locus (Wang and Chang, 2011; Huarte, 2015; Klinge, 2018). In addition, lncRNAs usually play a crucial role in cis- or trans-regulation of gene expression at their original sites or other locus in the genome. They also perform scaffolding function and remodeling chromatin through recruiting epigenetic complexes and ribonucleotide nucleotide proteins. The functions of other lncRNAs are executed *via* targeting mRNAs or regulating the post-transcriptional mechanism of genes. The subcellular locations of lncRNAs have a significant effect on their functional properties. The lncRNAs which are located in the nucleus can regulate genetic transcription and perform epigenetic modification by binding DNA to generate RNA–DNA triplex complex. As well, cytoplasmic lncRNAs can affect the stability of mRNAs and act on posttranscriptional regulation (Mercer and Mattick, 2013). For example, lncRNAs which are located in the nucleus can contribute to both RNA processing and protein modifications *via* interacting with RNA-binding proteins (RBPs) (He et al., 2019). In addition, they may contribute to up-regulated expression of mRNAs by acting as miRNA sponges and reduce the regulatory effects of miRNAs (Kinney and Pradhan, 2013; Militello et al., 2017). Also, they may be involved in protein synthesis or interact with other proteins and RNAs to influence cellular signaling cascades (Kinney and Pradhan, 2013; Sanchez Calle et al., 2018). LncRNAs can also generate a secondary and/or tertiary structure that provides multiple binding sites for proteins and other regulatory RNAs (Liu et al., 2017). For example, lncRNAs may bind DNA-binding proteins and prevent DNA-binding proteins from attaching to related transcription factors. A typical example is that some lncRNAs can stop DNMT1 from binding its targeted DNA. Thus, the methylation of the targeted DNA has been affected to some extent. As a result, transcriptional activation of the gene is influenced (Hung et al., 2011). As well, lncRNAs can play an important role in DNA damage response and cellular division (Hajjari et al., 2014). No matter the biological mechanism of lncRNAs, there is enough evidence that lncRNAs are involved in the numerous normal and abnormal cell functions.

## The Clinical Manifestations and Traditional Pathogenesis of Fragile X Syndrome

In essence, men are more likely to get FXS than women. This is because the X chromosome is linked to recessive inheritance. In fact, about 80% of male patients have intellectual disabilities. According to IQ, the severity is classified as mild, moderate, and severe mental disability, with corresponding IQ values at 40–54, 50–70, and less than 20, respectively. Many patients with full mutation are disabled moderately (Greco et al., 2002). They tend to suffer from midface hypoplasia, language barrier, autism, and macro-orchidism (Verkerk et al., 1991) while women are considered to be the carriers of FXS. More specifically, about 70% of women have normal intelligence as carriers, and female FXS patients only tend to have mild mental retardation. Female premutation is likely to suffer from FXPOI, leading to a reproductive decline.

The full mutation is responsible for FXS associated with inherited intellectual and developmental disability. With full mutation, the promoters and CpG islands of FMR1 gene are highly methylated. Meanwhile, the associated histone proteins are hyperacetylated and chromatin aggregated. Then, the silencing of FMR1 gene transcription is attributed to the absence of fragile X mental retardation protein (FMRP), a protein product encoded by the causative gene (Fu et al., 1991; Ali et al., 2017). The expression of FMRP begins at the early stage of development and lasts a lifetime. The expression of FMRP widely exists within all mammalian tissues. However, it is particularly abundant in the testis and brain. In the brain, FMRP exists mainly in the cytoplasm of neurons, including soma, dendrites, and synapses. FMRP, a kind of mRNA-binding protein, can associate with ribosomes and be involved in the aggregation of mRNA as well as regulation of the transcription efficiency of targeted genes. It also participates in protein synthesis of axons and dendrites. Therefore, the absence of FMRP might lead to the abnormal translation of mRNA and the abnormal structure and function of synapsis, which would affect the function of the nervous system (Pieretti et al., 1991; Sutcliffe et al., 1992; Darnell and Klann, 2013). The deduction supports that the deficient expression of FMRP is responsible for intellectual disability, thus patients exhibit a series of clinical manifestations (Weiler et al., 1997; Brown et al., 1998; Antar et al., 2005). Meanwhile, FMRP can potentially play an important role in the nucleus. Some existing researches have reported that FMRP may also be involved in mediating the DNA-damage response pathway through binding to methylated H3K79 chromatin (Liu et al., 2012).

## The Clinical Manifestations and Traditional Pathogenesis of Fragile X-Associated Tremor/Ataxia Syndrome and Fragile X-Related Primary Ovarian Insufficiency

### The Clinical Manifestations of Fragile X-Associated Tremor/Ataxia Syndrome and Fragile X-Related Primary Ovarian Insufficiency

Premature ovarian insufficiency (POI) is the cessation of ovarian function before 40 years of age. POI refers to the loss of germination and hormone function before normal physiological menopause as a result of the exhaustion of ovarian follicles (Hoek et al., 1997). As well, the risk of menstrual dysfunction, diminished ovarian reserve, and infertility is increased due to POI. In contrast, the age of menopause is decreased for POI patients. Laboratory tests have revealed that hypoestrogenism and elevated gonadotropin serum levels, which are characterized by low estradiol (E2) levels (<20 pg/ml), increased gonadotropin levels (follicle-stimulating hormone ([FSH] > 20 IU/l), low anti-Müllerian hormone (AMH) levels – <0.5 ng/ml (<1 ng/ml), and low inhibin B levels (Bellipanni et al., 2001). FSH levels may vary from cycle to cycle. Meanwhile, AMH is considered the best marker of ovarian reserve. As well, estrogen deficiency leads to the first symptoms: excessive sweating, tension, diminished libido, hot flushes, weakness, and mucous membrane dryness. In addition, substantial chronic hypoestrogenism may cause bone injuries and a higher risk of bone fracture. Even for younger women with POF, it is possible to have a consequent decrease in bone mineral density. Therefore, densitometry testing is necessary. Deficient estrogen levels are associated with metabolic disorders, thus can lead to cardiovascular diseases such as hypercholesterolaemia, atherosclerosis, and urogenital atrophy – infections and vaginal dryness (Fink et al., 2018). However, lower fertility or even infertility is one of the most troubling POF-associated problems for young women. However, for a majority of the women suffering from premature ovarian insufficiency (POI), etiology is still completely unknown. The risk of POI for premutation carriers with CGG repeat on one allele has been found to be high (up to 35%), while only 1% of women in the general population suffer from POI. As well, noncarriers might experience menopause 5 years later than premutation carriers, since these carriers’ ovarian function is obviously destroyed (Bretherick et al., 2005; Bodega et al., 2006; Mailick et al., 2014). Recent studies have demonstrated that overt premature ovarian insufficiency is correlated with premutation repeat lengths. Also, there is a linear relationship between the size of CGG trinucleotide repeats and the risk of POI/ovarian phenotype. With an increase in the size of repeats, the risk of POI elevates and reaches a plateau, but decreases beyond 80–100 repeats (Sullivan et al., 2005; Ennis et al., 2006; Tejada et al., 2008). Allen and colleagues realized that the average age of menopause tends to decline in all premutation carriers, which is apparently the medium-sized repeats with the lowest menopause age. It means that medium-sized repeats have lower odds ratio for fertility and an increased rate of dizygotic twinning compared with normal individuals while the rate of spontaneous abortion does not increase. This indicates that it puts no damage on the quality of oocyte. Both low and high repeats tended to suffer the same experience from insufficient ovarian reserve, although not as serious as medium-sized carriers (Allen et al., 2007).

FXTAS is a neurodegenerative disorder characterized by progressive intention tremor, gait ataxia, psychiatric symptoms, parkinsonism, cognitive decline, and autonomic disorders, which typically occurs after 50 years of age (Hagerman et al., 2001; Jacquemont et al., 2003; Kong et al., 2017). Main principal neuropathological features of FXTAS include Purkinje cell loss, brain atrophy, and ubiquitin-positive intranuclear inclusions which are mostly present in single, large (~2–5 μm of diameter), and spherical aggregates (Weiler et al., 1997; Greco et al., 2002, 2007; Jacquemont et al., 2003). These aggregates exist in different areas of the brain, especially in the hippocampus, and they are also found in the brain’s Purkinje cells. Additionally, rare intranuclear inclusions, which are ubiquitin-positive and contain various chaperones, are also detected in the tissues outside of the central nervous system (Iwahashi et al., 2006). In particular, FXTAS is more common in males than in females (Rodriguez-Revenga et al., 2009). Previous studies have found that FMR1 premutation can contribute to FXTAS development (Garcia-Arocena and Hagerman, 2010). As well, female permutation carriers do not suffer from FXTAS in most cases due to the defense mechanism that the pathogenic mutant, allele (located at X-chromosome) may become inactivated randomly. In addition, it affects an estimated 46% of males and 17% of females. Compared to carriers of the CGG premutation allele with control (<30) CGG repeat size, the FMR1 mRNA level of the former has increased 2 to 8-folds *via* brain and blood analysis (Tassone et al., 2000, 2004). Tassone and colleagues attributed the higher FMR1 mRNA level to an increased transcriptional activity of the FMR1 gene (Tassone et al., 2007), which may be due to epigenetic modifications in the result of CGG repeat expansion itself (Todd et al., 2010; Usdin and Kumari, 2015).

### The Traditional Pathogenesis of Fragile X-Associated Tremor/Ataxia Syndrome and Fragile X-Related Primary Ovarian Insufficiency

However, little is known about the mechanisms that contribute to the development of FXPOI and FXTAS. These individuals with an allele of an expansion beyond 200 CGC trinucleotide repeats lead to the incomplete absence of FMRP, but they still perform their function normally. Thus, insufficient FMRP does not submit to be the culprit of developing FXPOI/FXTAS (Sherman et al., 2014). Kenneson put forward that FMR1 RNAs’ transcription of the permutation carriers was positively relevant with the size of CGG trinucleotide repeats while the FMRP translation was negatively correlated with the size of CGG trinucleotide repeats (Kenneson et al., 2001). The excessive FMR1 mRNAs in premutation carriers contribute to several proteins’ dysregulation and deposition. Along with FMR1 mRNAs, these proteins are present in several parts of the body in the form of cell inclusions, which is located in CNS, peripheral nervous system (particularly autonomic ganglia), pituitary, and Leydig cells (Greco et al., 2006; Gokden et al., 2009). Therefore, partial FMRP deficiency and/or RNA toxicity may be involved in the pathogenic mechanism of FXPOI/FXTAS. In addition, abnormal translation of the CGG repeats can result in the production of polyalanine (FMRpolyA). The polyglycine (FMRpolyA) and other polypeptides containing proteins are neurotoxic (Hall and Berry-Kravis, 2018). FMRpolyG proteins can be detected in the brains and other tissues of individuals, but FMRpolyA could only be demonstrated in transfected cells with FXTAS. The phenomenon illustrated that FMRpolyA may contribute to the development of FXTAS (Hukema et al., 2015).

The theory suggests that dynamic intranuclear long rCGG RNA, translated by the gene with CGG trinucleotide repeats, can contribute to making normal cells dead and nonfunctional *via* tracking and binding to a wide range of RNA-binding proteins (RBP). The intranuclear long rCGG RNA is sequesters-specific and its sequestration can make the viability of normal cells decreased (Sellier et al., 2010). Also, the phenomenon is confirmed in another study, suggesting the fully mutated carriers or those with 4,199 methylated repeated alleles lead to silencing the expression of FMR1 gene, but did not suffer from FXPOI or FXTAS. That leads to the conclusion that the silence of disease-causing gene (FMR1) and protein products (FMRP) is not the main culprit. On the contrary, FMR1 transcript levels increase with premutation carriers. In addition, Elizur et al. have confirmed the fact that FMR1 mRNAs of both male and female premutation carriers are up-regulated, and FMR1 mRNAs play a significant role in FXTAS and FXPOI (Elizur et al., 2014).

## Categories of Pathogenic Long Noncoding RNAs Originating From FMR1 Gene

**Figure 1:**
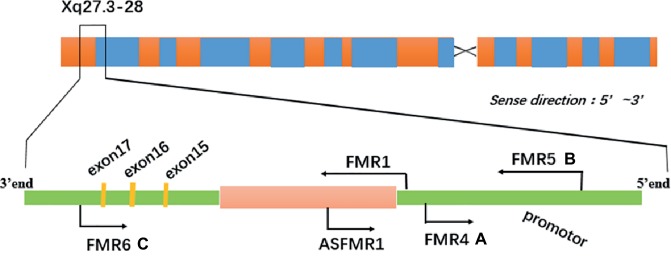
The transcriptional landscape of the FMR1 gene locus is complicated **(A)** FMR4 is transcribed upstream of FMR1 in the antisense direction. **(B)** FMR5 is a sense-oriented transcript from the FMR1 promoter and its transcription start site (TSS) is situated 1 kb upstream from FMR1 TSS. **(C)** FMR6 is transcribed in the antisense direction from the 3′ UTR of FMR1 overlapping exons 15–17.

Previous studies have indicated that lncRNAs contribute to the pathogenesis of both permutation and full mutation carriers, especially nervous disorders. In addition, it is reported that the expression of lncRNAs is different, and several relevant lncRNAs originate from the FMR1 gene locus in both FXS patients and premutation carriers. This suggests that they may be markers to diagnose or evaluate relevant disorders (Figure 1; Ladd et al., 2007; Khalil et al., 2008). And the discovered lncRNAs originating from FMR1, including FMR4, FMR5, and FMR6 as shown in Figure 1. Additionally, BC1 RNA and lncRNA TUG1 which originate from other genes also contribute to making permutation carriers sick.

### FMR4 Can Affect Cell Proliferation or Differentiation

FMR4, an untranslated primate-specific lncRNA (2.4 kb), is transcribed upstream of FMR1 in the antisense direction. It is widely expressed in body development. In particular, FMR4 has been widely detected during our growing up years. Also, the expression of FMR4 exists in some adult tissues such as brain, small intestine, spleen, colon, liver, and placenta except in the ovaries, prostate, pancreas, or testes (Hinds et al., 1993). During embryonic and/or fetal development, FMR4 is likely to express in these orangs and tissues (ovaries, prostate, pancreas, or testes). Similarly, the expression of FMR4 is detected highly in kidney and heart of fetus. Considering that a number of people suffering from FXS have cardiac dysfunctions, such as prolapse of mitral valve and aortic root dilation, FMR4 which expresses highly in heart may have a functional role to play in the relevant pathogenic mechanism (Sreeram et al., 1989).

**Figure 2:**
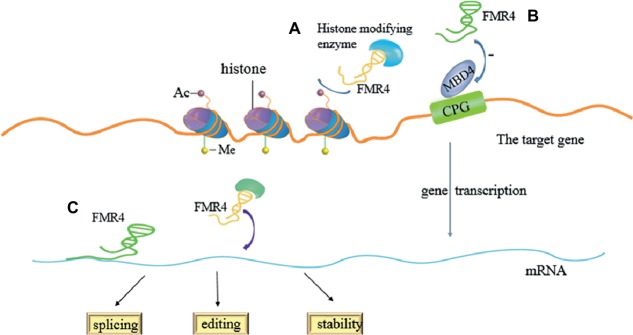
Regulation of FMR4 in a variety of ways. **(A)** FMR4 binds histone-modifying enzymes to develop complexes which can regulate transcription of the target gene. **(B)** FMR4 can play role in DNA mismatch repair, transcriptional inhibition, and apoptosis regulation as a negatively regulating factor of MBD4. **(C)** FMR4 can induce the changes of transcriptional levels by directly aiming at mRNAs splicing, editing, or stability, or by forming scaffolds for the recruitment of functional protein.

FMR4, a product of RNA polymerase II, can be detected in normal people as well as in premutation carriers, but not in FXS with full mutation. It also has similar half-life to FMR1 mRNA. Similar to FMR1 mRNA level, the expression of FMR4 is up-regulated in premutation carriers and silenced in brain tissue of full mutation carriers (FXS). Some previous researches have shown that overexpression and knockdown of FMR4 can alter the expression of these genes, which has an effect on cellular proliferation or differentiation. As a chromatographic transcript, FMR4 can induce the changes of transcriptional levels by directly aiming at mRNAs splicing, editing, or stability, and by binding histone-modifying enzymes to develop complexes. However, it is not certain whether these observed results are affected by epigenetic changes, RNA-protein interactions, or downstream effects. The study also confirmed the methyl-CpG-binding domain protein 4 (MBD4), which is a negatively responsive gene of FMR4. MBD4 is a member of MBD nucleoprotein family, and it has two domains: one is methyl-CpG-binding domain which can specifically bind methylated CpG, and the other one is a DNA glycosylase domain which is associated with the catalytic activity. Thus, MBD4 is crucial in DNA mismatch repair, inhibition of transcription, and the regulation of apoptosis (Yakovlev et al., 2017). In addition, the study revealed that FMR4 very likely shares a bidirectional promoter with FMR1. The expression of FMR4 is developmentally regulated and shows negative relation to the expression of both FMR1 and MBD4 in human neural precursor cells, which are in differentiation. Therefore, all evidence implicates that as a kind of LncRNAs and FMR4 can regulate the function of relevant genes which can regulate gene, and the transcript may act in cellular development (Peschansky et al., 2015). However, Peschansky and colleagues also suggested that the genes regulated by FMR4 enrich and are involved in cell proliferation and neural development. S-phase marker assays further exhibited that FMR4 may up-regulated cell proliferation, rather than differentiation of human neural precursor cells (hNPCs). They confirmed the theory by using transfection, qPCR, and subcellular fractionation, and other technologies. The experimental group is that HEK293T cells are transfected with pcDNA3.1-FMR4, while the control group is that cells are transfected with empty pcDNA3.1 control vector or silencer negative control siRNA aiming at FMR4. And FMR4 is highly expressed in the experimental group but not in the control group. They found that the silencing and overexpression of FMR4 can lead to the genome-wide change in histone methylation. The situation also exists in other mRNAs which have been confirmed to act in developmental or neurophysiological roles. FMR4 works mainly by forming scaffolds for the recruitment of histone-modifying complexes or other proteins to affect the stability, splicing, or editing of relevant mRNAs (Figure 2; Peschansky et al., 2016).

The phenomenon was also found in the research of Ahmad M and colleagues. As mentioned above, there are no overlaps between the FMR4 and FMR1 because FMR4 lies in the upstream of FMR1 with 2.4 kb in the antisense direction. Thus, they used relevant siRNAs to knockout FMR1 and found that there has been no change in the expression of FMR4. Thus, they reported that expression of FMR4 cannot be affected by FMR1. These observations have indicated that FMR4 is not directly derived from regulatory transcript for FMR1. But siRNA knockdown of FMR4 can cause an increase of cell apoptosis and the changes of cell cycle. In addition, they simultaneously found that the overexpression of FMR4 resulted in an increased proliferation *in vitro*. Thus, FMR4 has a significant effect on cell proliferation *in vitro*. The evidence implicated that FMR4 can act at anti-apoptosis in HeLa cells and HEK293T and suggests that studying genomic locus can make us discover unknown functions of the gene. They also speculate that changes in the expression of FMR4 may affect the clinical manifestations of FXS or associated disorders (Khalil et al., 2008).

Another transcript, ASFMR1, originates from the CGG expanded repeats in the 5′ UTR of FMR1, and was reported by Ladd and colleagues. The expression of ASFMRI is silenced in full carriers and increased in premutation carriers, with the changes being similar to FMR4. Ladd and colleagues also evaluated that FMR4 is likely to be nested in the 3′ UTR of ASFMR1 due to its one splice variant overlapping that of FMR4. Besides, ASFMR1 is widely expressed in human tissues with relatively high expression in brain. The ASFMR1 transcript is transported to the cytoplasm and contains a potential proline-rich ORF, indicating that ASFMR1 has a conserved cellular function and can potentially be associated with FXS and FXTAS (Ladd et al., 2007).

### FMR5 Is a Kind of Sense-Oriented Long Noncoding RNA

FMR5 is a sense-oriented lncRNA and its transcription begins around 1 kb upstream from the FMR1 transcription start site (TSS) which overlaps with the FMR1 promoter. Meanwhile, FMR5 and FMR6 are discovered by using a new technology called “Deep-RACE.” This technology can combine next-generation sequencing with rapid amplification of cDNA ends (RACE). The expression of FMR5 reportedly appeared in some brain tissue with full mutation, permutation carriers, and normal individuals (Pastori et al., 2014). There are similar expression levels of FMR5 in brain tissue with normal people, full mutation, and permutation carriers, which shows that FMR5 transcription is not related to chromatin methylated modifications. Kumari and Usdin also indicated that the transcription of low-riched transcripts such as FMR5 may be repressed by the essence of negative histone marks in the FMR1 locus. This is consistent with the discovery that trimethylation of histone H4 at lysine 20 (H4K20me3) as a negative chromatin mark as well as trimethylation of histone H3 at lysine 9 (H3K9me3) are related to exon 1 of the FMR1 gene, such as CGG expanded repeats, but not connected with the promoter region (Kumari and Usdin, 2010). At the same time, lower levels of three positive chromatin marks, including H3K4 dimethylation (H3K4me2), H3 acetylation (H3Ac), and H4 acetylation (H4Ac) combine with the FMR1 promoter in full mutation carriers (Gheldof et al., 2006). Kumari and Usdin suggested negative histone modifications on silenced FMR1 may contribute to developing FXS because these modifications enrich FX alleles, and the intrinsic and local repeats may lead to the silence of FMR1 (Kumari and Usdin, 2010).

### FMR6 Regulates Translational Efficiency and/or Stability of FMR1

The expression of FMR5 and that of FMR6 are dependent on completely different patterns. FMR6, a spliced lncRNA, is transcribed by the 3′ UTR of FMR1 overlapping exons 15–17 in the antisense direction, and FMR6 may combine with the FMR1 mRNA because it is complementary to the 3′ region of FMR1. A study found that selective siRNA aiming to knockdown the nonoverlapping regions of the β-secretase-1 antisense transcript (BACE1-AS) made the expression of β-secretase-1 (BACE1) mRNA and protein decreased, which indicated that BACE1-AS can regulate BACE1 mRNA and BACE1 protein expression subsequently (Faghihi et al., 2008). And it follows that FMR6 may also regulate the splicing and stability of FMR1 mRNA and the expression level of FMRP. As well, FMR6 overlaps two microRNA binding sites, including miR-19a and miR-19b located in the 3′ UTR of FMR1, and the lncRNA may regulate translational efficiency or stability of FMR1 mRNA by the another way of combining with microRNAs (Pastori et al., 2014).

The expression of FMR6 is down-regulated in brain tissue from premutation carriers and fragile X patients (Pastori et al., 2014). Meanwhile, Shai E. Elizur and colleagues carried out a study to evaluate whether the accumulation of lncRNAs contributes to developing FXPOI. Their research suggested that FMR6 is expressed in ovarian granular cells from both premutation carriers and fragile X patients similar to FMR1 mRNA, and there is a marked nonlinearity between the FMR6 level of ovarian granular cells and the size of CGG repeats. Females in the medium-range CGG repeats (80–120) obviously keep up with higher FMR6 levels of granulosa cells. In addition, the transcription level of FMR6 is negatively associated with the number of oocytes detected. These findings indicate that in ovary granulosa cells of females with FXPOI, the accumulation of both FMR6 and FMR1 except FMR4, may result in ovarian dysfunction. Although the FMR1 premutation can result in primary ovarian insufficiency, the relationship is nonlinear but still not exact between ovarian reserve and CGG repeat numbers. We can speculate FXPOI is the result of an increased accumulation of FMR1 and FMR6 lncRNAs in ovarian granular cells in the intermediate range (80–120 CGG repeat). Also, more well-studied observations are required to explore the exact mechanism by which FMR1 mRNA and FMR6 contribute to FXPOI, and estimate whether these research findings might also be extended to the normal-range CGG repeats (Elizur et al., 2016).

## The FMRP-BC1-mRNA Inhibitory Complex

Presynaptic localization of FMRP has been confirmed in the CNS. FMRP can regulate the development of both axon and dendrite. Meanwhile, methylation of FMR1 gene two-way promoter can regulate the expression of lncRNAs. In addition, FMRP, a kind of protein involved in regulating the efficiency of translation and transporting messenger ribonucleoprotein (mRNP), can combine with the dendritic brain cytoplasmic RNA 1 (ncRNA BC1) to form the FMRP-BC1 complex. This complex can inhibit the translation of a certain subset of FMRP-targeted mRNAs in neurons.

**Figure 3:**
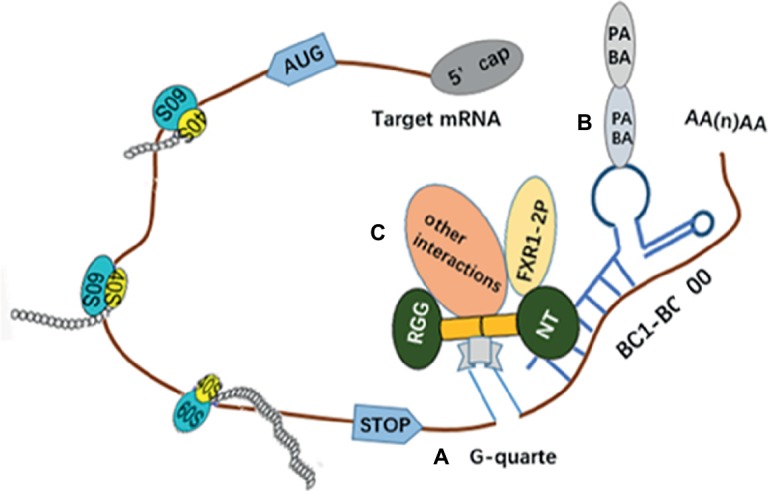
It is an illustration of how the BC2/200-FMRP complex recognizes and inhibits the translation of mRNAs. **(A)** The NT of FMRP can bind to the targeted mRNAs simultaneously *via* five regions of the longer stem loop of BC1 RNAs and G-quarter, and it would repress steadily the translation of the targeted mRNAs. **(B)** Poly(A)-binding protein (PABP) can connect with BC1 that actually act on the poly(A) tail of targeted mRNAs. **(C)** Other interactions also play a role in preventing the targeted mRNAs being.

BC1 ncRNAs have been regarded as negative translation regulators. Knockdown of BC1 RNAs’ expression can lead to remarkably increased neuronal excitability and epilepsy (Zhong et al., 2009). The BC1 RNA can also play a role as an adaptor molecule to connect several mRNAs with FMRP (Zalfa et al., 2005). BC200, BC1 analog in primates, can have more advantages compared with BC1 because its RNA distribution is also able to indicate the localization of dendrites and neuron-specific expression (Tiedge et al., 1993). Also, the FMRP have exhibited enough binding sites as a regulatory and transport factor, such as two evidently characterized KH domains which can bind RNAs, and the N and C termini which have affinity for RNA. However, only the RGG box of FMRP can only bind RNA with special sequence and/or structure. Similarly, G quartet is a kind of RNA with rich G. It can directly bind FMRP, recognize and connect with the above four domains (Darnell et al., 2001; Schaeffer et al., 2001). Also, a new RNA-binding motif originating from the N terminus (NT) of FMRP has been identified to be capable of binding to BC200 specifically and directly. In addition, the FMRP-BC1/BC200 complex can cover up the signal which can regulate the FMRP cycle in and out of the nucleus. Thus, the transportation of mRNP is affected and the relevant proteins cannot be transported into the nucleus, while the breakdown of the complex permits proteins to reenter the nucleus freely (Zalfa et al., 2005). Studies *in vitro* have indicated that BC1 can play its functional inhibition role by binding both PABA and eIF4A (the translational initiation factor) (Wang et al., 2002). Meanwhile, Lacoux C and colleagues proposed that the interaction between BC1 and FMRP may be regulated by 2′-O-methylation. They demonstrated that BC1 RNAs in neurons are 2′-O-methylated differentially and have an effect in binding FMRP to the complex (Figure 3; Lacoux et al., 2012).

## Long Noncoding RNA TUG1 Regulate Axonal Development *Via* Modulating SnoN-CCD1 Pathway

Current evidence confirms that lncRNA TUG1 has an obvious and close relationship with FMRP for patients suffering from cancer. Guo and colleagues pointed out that the transcription of FMRP is successfully suppressed in neurons transfected by Fmr1shRNA. They found out that the length and complexity of the dendrites were all reduced in the neurons whose FMRP expression was deficient compared with the control group whose FMRP expression was normal. As such, the data can confirm that FMRP plays a crucial role in axonal development. However, there was no significant change in the length and complexity of the dendrites in TUG1-deficient neurons transfected by TUG1 shRNA. The researchers found that down-regulated TUG1 expression slightly led to developing axon better in neurons and up-regulated TUG1 expression results in significantly shortening the axonal length. Meanwhile, FMRP deficiency led to overexpression of TUG1 and knockdown of TUG1 expression can repair the defects of axonal development in FMRP-deficient neurons. It indicates that TUG1 may interact with FMRP to specifically regulate axonal development of neurons. At the same time, they found that TUG1 can regulate axonal development by interfering with the SnoN-Ccd1 pathway, which is known to be involved in the development of axons. In addition, the reduced length of axon due to TUG1 up-regulation and FMRP deficiency can be rescued by the overexpression of Ccd1. However, making FMR1 silenced and TUG1 overexpressed does not alter the whole protein expression level of SnoN. It demonstrates that the interaction between TUG1 and FMRP regulates SnoN activity that would not be dependent on the ubiquitin-proteasome system. The system has been proved to be capable of activating SnoN pathway because the ubiquitin ligase Cdh1/APC can accelerate SnoN ubiquitination and subsequent degradation, and can consequently inhibit axonal development (Konishi et al., 2004; Guo et al., 2018).

It has been known that lncRNAs can affect gene expression level *via* binding to specific transcriptional factors and inhibit or enhance the activity of these specific transcriptional factors. Similarly, TUG1 can also inhibit the transcriptional activity of SnoN by binding with SnoN, which would lead to a decreased expression of Ccd1, a well-known SnoN-targeted gene. Therefore, these evidence provide another potential mechanism of how lncRNA TUG1 can regulate the transcriptional activity of SnoN. In other words, lncRNAs can also combine with particular transcriptional factors to repress or promote the activities of these transcriptional factors, thus regulate the expression of relevant genes, just as TUG1 can bind with SnoN to inhibit its transcriptional activity. This will result in the down-regulated expression of Ccd1, known as SnoN-targeted gene (Geisler and Coller, 2013). A large number of previous studies have proved that lncRNA TUG1 is closely related to FMRP for patients suffering from cancer. However, whether the relationship between lncRNA TUG1 and FMRP for patients suffering from FXS and FXPOI/FXTAS is the same or not, there is a need for more in-depth studies to verify it. If the answer is yes, then it could open new avenues of research and treatment for FXS and FXPOI/FXTAS.

## Conclusion and Perspective

The expression of FMR6, FMR5, FMR4, and FMR1 is different in patients’ brain tissues with FXS and FXTAS/FXPOI, which could probably be as a result of the differences in CGG trinucleotide repeat numbers, DNA methylation degree, or histone modification degree. FMR4, a product of RNA polymerase II, indicated increased expression in premutation carriers and silenced expression in full mutation carriers. It can also regulate the expression of relevant genes during differentiation of human neural precursor cells (Kumari and Usdin, 2010). Meanwhile, both FMR5 and FMR6 are new transcripts from the FMR1 gene locus. The expression of FMR5 appears in some human brain regions in both FXS patients and premutation carriers, while the expression of FMR6 is silenced in premutation and full mutation carriers. For FXS and FXTAS/FXPOI patients, it is feasible that the levels of these transcripts (FMR6, FMR5, FMR4 and FMR1) can correspond to different clinical manifestations and results. Therefore, these lncRNAs may be taken for markers to diagnose and evaluate FXS and FXTAS/FXPOI. And there is a need for additional studies to evaluate whether any undiscovered functional properties of each transcript may result in clinical phenotypes of FXS and FXTAS/FXPOI patients (Pastori et al., 2014). Most importantly, several studies have suggested that FMRP also has a direct interaction with BC1, which affects its functional regulation and transportation. As well, lncRNA TUG1 can bind it to decrease its stability. In addition, lncRNA TUG1 can regulate axonal development by combining with SnoN and mediating SnoN-Ccd1 pathway. Also, there is a need to deeply explore an alternative potential mechanism of modulating the transcriptional activity of SnoN by lncRNA TUG1 and the function of lncRNA TUG1 in patients suffering from FXS and FXPOI/FXTAS (Fabian and Sonenberg, 2012). LncRNAs can be considered a new biomarker for human disease. It has been applied to new diagnostic or prognostic markers in bodily fluid samples, such as urine samples of patients suffering from cancer to detect the lncRNA prostate cancer antigen 3. The lncRNA can improve diagnosis of prostate cancer (Reis and Verjovski-Almeida, 2012). Similarly, to determine whether the lncRNAs’ expression levels are related to clinical manifestations of fragile X Syndrome, it is necessary to detect these transcripts in a large number of patients. In particular, blood samples provide the most practical choice for both experimental and potential prognostic purposes. By understanding the mystery of these lncRNAs, the diagnosis and treatment of FXS, along with its associated disorders, may be more accurate and effective in the future.

## Author Contributions

GH and HZ wrote the manuscript, carried out image analysis, and contributed to this work equally. SW drew the figures. SW and MC co-supervised GH and HZ, participated in discussions, and commented on the manuscript. TX conceived the idea, directed, and critically reviewed the manuscript.

### Conflict of Interest Statement

The authors declare that the research was conducted in the absence of any commercial or financial relationships that could be construed as a potential conflict of interest.
